# Fluoroscopically guided transforaminal epidural dry needling for lumbar spinal stenosis using a specially designed needle

**DOI:** 10.1186/1471-2474-11-180

**Published:** 2010-08-11

**Authors:** Kang Ahn, Hyung-Joon Jhun, Tae-Kyun Lim, Yong-Seung Lee

**Affiliations:** 1 Chronic Pain Management Centre, Cha Biomedical Centre, Kangnam Cha Hospital, Cha University, 605, Yeoksam-Dong, Kangnam-Ku, Seoul, Republic of Korea; 2 Centre for Interventional Pain Management, Daejeon Woori Hospital, 1419, Metrozone, Doonsan-Dong, Seo-Ku, Daejeon, 302-531, Republic of Korea

## Abstract

**Background:**

This report describes the methodological approach and clinical application of a minimally invasive intervention to treat lumbar spinal stenosis (LSS).

**Methods:**

Thirty-four patients with LSS underwent fluoroscopically guided transforaminal epidural dry needling using a specially designed flexed Round Needle. The needle was inserted 8-12 cm lateral to the midline at the level of the stenosis and advanced to a position between the anterior side of the facet joint and pedicle up to the outer-third of the pedicle. The needle was advanced medially and backed laterally within a few millimetres along the canal side of the inferior articular process between the facet joint and pedicle. The procedure was completed when a marked reduction in resistance was felt at the tip of the needle. The procedure was performed bilaterally at the level of the stenosis.

**Results:**

The average follow-up period was 12.9 ± 1.1 months. The visual analogue scale (VAS) pain score was reduced from 7.3 ± 2.0 to 4.6 ± 2.5 points, the Oswestry Disability Index (ODI) score decreased from 41.4 ± 17.2 to 25.5 ± 12.6% and the average self-rated improvement was 52.6 ± 33.1%. The VAS scores indicated that 14 (41.2%) patients reported a "good" to "excellent" treatment response, while 11 (32.4%) had a "good" to "excellent" treatment response on the ODI and 22 (64.7%) had a "good" to "excellent" treatment response on the self-rated improvement scale.

**Conclusions:**

These results suggest that fluoroscopically guided transforaminal epidural dry needling is effective for managing LSS.

## Background

Lumbar spinal stenosis (LSS) is a painful and potentially disabling condition that is defined as a narrowing of the lumbar spinal canal, nerve root canal or intervertebral foramina. It is often encountered in the geriatric population. The primary causes of spinal canal constriction are a protruding intervertebral disc, hypertrophied facet joint and thickened ligamentum flavum [1].

Patients suffering from LSS develop pain, paraesthesias, numbness and weakness in the back and legs caused by compression of the lumbosacral nerve roots in the constricted neural canal and foramina [2]. Neurogenic claudication, the most common symptom of LSS, is the progressive onset of radicular pain, paraesthesias and numbness and may cause weakness during walking or be worsened by walking. LSS is exacerbated by lumbar extension and improves with lumbar flexion [3].

A wide variety of conservative methods has been developed to treat LSS. They include medication, physical therapy, behavioural therapy, orthopaedic devices, girdles, acupuncture and manual therapy. Patients refractory to conservative therapy generally undergo surgery to decompress the spinal canal and the neural foramina, and eliminate pressure on the spinal nerve roots [4,5].

We developed a minimally invasive interventional technique to treat LSS. The present study illustrates the methodological approach and clinical application of this technique.

## Methods

### Subjects

The subjects were 34 consecutive patients (9 men and 25 women) with LSS who underwent fluoroscopically guided transforaminal epidural dry needling at a chronic-pain management centre in Korea in 2008. Patients included in the study had received at least one treatment session and 12 or more months had elapsed since their last session.

The diagnosis of LSS was made based on clinical history, physical examination and three-dimensional computed tomography (3-D CT) findings. The inclusion criteria were back pain and/or radiating pain to the lower leg, accompanied by neurogenic claudication, exacerbated by lumbar extension and relieved by lumbar flexion; altered physical findings corresponding to chronic neuropathic pain and spinal stenosis, such as localised vasoconstriction, hair loss or trophoedema, limitation of extension and the presence of lumbar tenderness [6,7], and a reduction in the cross-sectional area of the spinal canal and neural foramina accompanied by degenerative changes of the lumbar spine, such as disk protrusion, hypertrophy of the ligamentum flavum, hypertrophy of the facet joints or the appearance of osteophytes on the 3-D CT. Studies have reported a high prevalence of abnormalities on imaging, including spinal stenosis in asymptomatic subjects and mismatching between symptoms and image findings [8,9]. Therefore, we evaluated whether the CT findings of individual lumbar spinal levels sufficiently corresponded to the patient's history and physical findings when determining treatment level(s). If a patient had symptoms corresponding to LSS and altered physical and abnormal CT findings were detected simultaneously for a specific lumbar joint, we deemed that level the treatment target of the intervention.

All of the patients were provided with comprehensive information on the benefits and potential risks (such as infection, bleeding, post-needling soreness and dural puncture) of the intervention and gave informed consent prior to the treatment. A follow-up evaluation was conducted at hospital 3 weeks after the treatment. If a patient complained of persistent pain or limited improvement, the treatment was repeated at 3-week intervals.

As this study was a follow-up of patients who had previously received the intervention, written informed consent was not required. The institutional review board approved the study protocol.

### Needle and procedure

**Figure 1:**
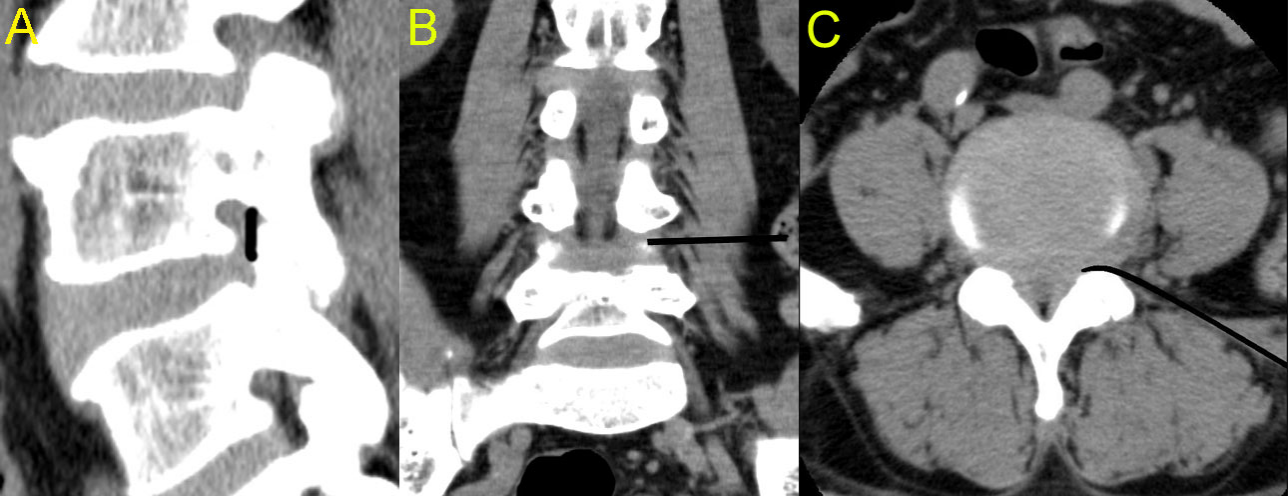
**Three-dimensional CT images of the transforaminal epidural dry needling procedure**. The sagittal CT image shows the area where the transforaminal epidural dry needling was performed (A). The coronal (B) and axial (C) CT images show the depth and route of needle insertion.

Many studies have demonstrated the anatomy of the lumbar spine [10] and the pathophysiological mechanisms of LSS [11-13]. Based on these studies, we postulated that the posterior epidural space adjacent to the intervertebral foramen that lies between the anterior side of the facet joint and pedicle in the sagittal plane (Figure 1A), usually up to the outer-third of the pedicle in the coronal plane (Figure 1B) and between the ligamentum flavum and the dural sleeve in the axial plane (Figure 1C) should be the primary treatment target in LSS. However, additional treatment was performed in the anterior epidural space if the posterior epidural approach did not produce a favourable treatment outcome.

**Figure 2:**
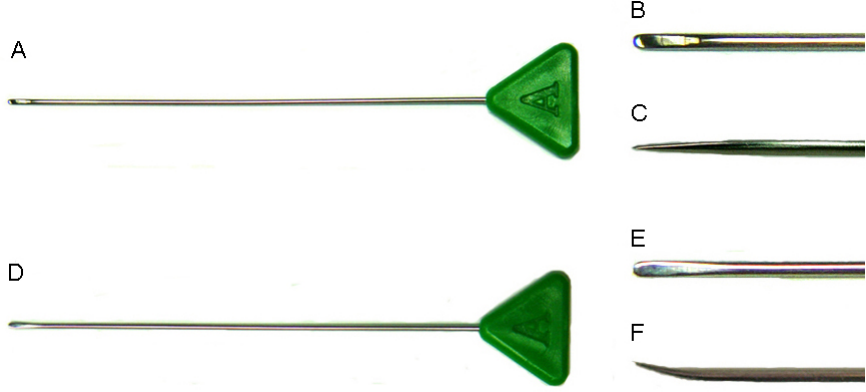
**The needle used in fluoroscopically guided transforaminal epidural dry needling to treat lumbar spinal stenosis**. The Round Needle before bending (A) and close-ups of its tip from above (B) and the side (C). The flexed Round Needle after bending (D) and close-ups of its tip from above (E) and the side (F).

We treated LSS using a specially designed needle (Round Needle^® ^Hansung Precision, Korea) that is 1.2 mm in diameter and 120 mm long (Figure 2A). The needle is streamlined, solid and flexible, and has a blunt, round tip [14] (Figures 2B and 2C). We bent the needle 5-30° at a point 2-4 mm from the tip (Figures 2D, E and 2F) using surgical tweezers to allow it to accurately reach the target structures and avoid damage to the spine, particularly possible damage to arteries because of anatomical variation or neovascularisation of the lesion [15]. The degree of bending was determined after identifying the shape of the intervertebral foramen on the axial CT image.

The patients were instructed to lie on a table in the prone position. A C-arm fluoroscope was used to identify the bony landmark in the anteroposterior view and the skin was marked and cleaned. Local anaesthesia was achieved with 1% lidocaine; however, if patients experienced severe pain or became irritable during the procedure, they were sedated with intravenous midazolam or propofol.

**Figure 3:**
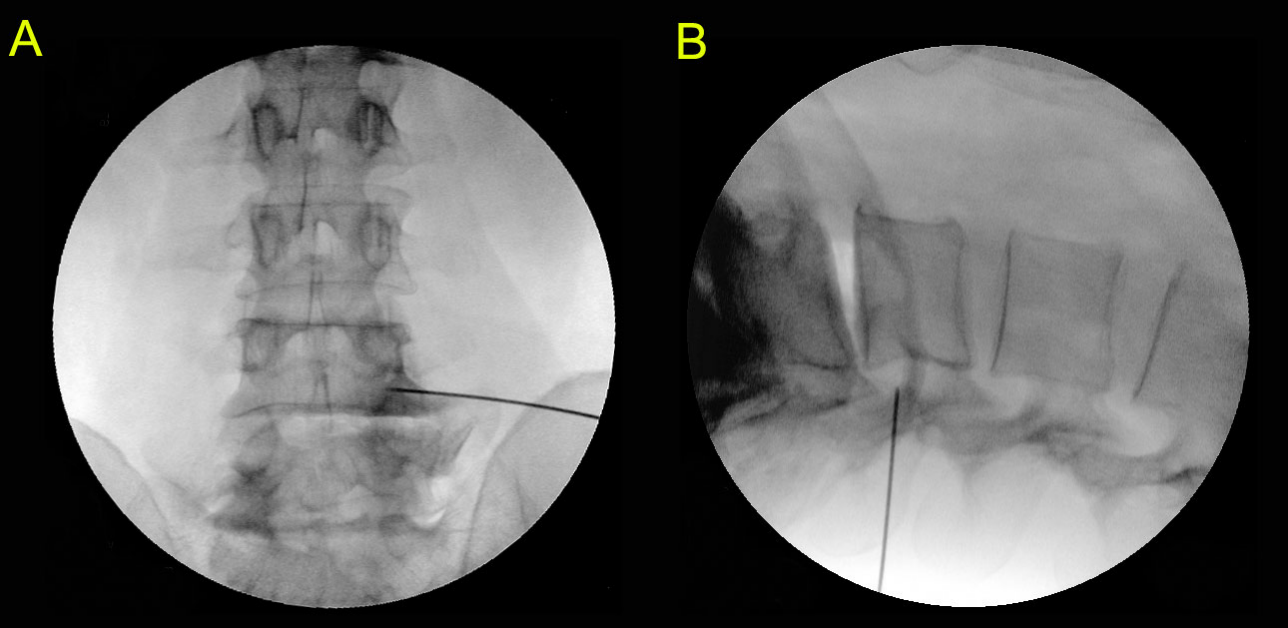
**Fluoroscopy images of the transforaminal epidural dry needling procedure**. The anteroposterior (A) and lateral (B) fluoroscopy images are from a patient undergoing the intervention.

At the level of the stenosis, the flexed Round Needle was inserted 8-12 cm lateral to the midline with the concave surface facing up. The needle was advanced at a 15-30° angle to the horizontal plane until the flexed tip contacted the lumbar spine (Figure 3A). Then the C-arm was turned to the lateral view. The tip of the needle was positioned between the anterior side of the facet joint and pedicle (Figure 3B), and was then advanced to the outer-third of the pedicle (Figure 1B). It was advanced further, to the inner line of pedicle, as an additional treatment for the anterior epidural space if the previous posterior epidural approach did not produce a favourable treatment outcome. The needle was advanced medially and backed laterally a few millimetres along the canal side of the inferior articular process between the anterior side of the facet joint and pedicle. The procedure was complete when we felt a marked reduction in resistance at the tip of the needle. The procedure was performed bilaterally at each level of the stenosis (Additional file 1: A movie demonstrating the technique).

### Outcome measures

We selected three outcome measures to evaluate the effectiveness of the treatment: a self-rated pain score, the Oswestry Disability Index (ODI) and self-rated improvement following the intervention. The self-rated pain score and the ODI were the primary outcomes and were assessed before and after treatment. Self-rated improvement was a secondary measure and assessed only after treatment.

The patients were asked to rate their pain level using a visual analogue scale (VAS) that consisted of a 10-cm line anchored by two extremes. The ODI is one of the most common condition-specific outcome measures used for the management of spinal disorders [16]. It consists of 10 sections related to back pain and level of function: pain intensity, personal care, lifting, walking, sitting, standing, sleeping, sex life, social life and travelling. In each section, the patient is asked to choose the statement that best describes his/her status. The statements are scored from 0 to 5, corresponding to the level of limitation; 0 indicates the least limitation and 5 indicates the greatest limitation in each category. The total score can range from 0 (highest level of function) to 50 (lowest level of function). Some patients did not respond to every section; thus, the per cent disability was calculated on the basis of the total possible points. For example, if all 10 sections were completed, the total per cent disability was calculated as (total points/50) × 100. If one section was missed or not applicable, the per cent disability was calculated as (total points/45) × 100 [17].

The pre-interventional baseline data were obtained using a self-administered questionnaire when the patients initially visited hospital. The post-interventional follow-up data were obtained using a telephone survey. The follow-up outcome measures consisted of 12 items. It took 5-10 minutes to respond to all items. Medical research, including a follow-up study of musculoskeletal intervention, occasionally uses telephone surveys to obtain data, and arguments against the validity of such methods have not been raised [18]. To ensure the validity of the follow-up data, an independent researcher conducted the survey using a structured questionnaire in a standardised manner.

The Wilcoxon signed-rank test was used to evaluate the differences between pre- and post-intervention self-rated pain and per cent disability on the ODI scores. The null hypothesis of no improvement after treatment was tested to assess self-rated improvement following treatment. Additionally, we evaluated the per cent change of the individual outcome measures. The self-rated improvement score and the per cent change on the VAS and ODI scores were defined as follows: ≥ 75% was "excellent", a response of 50-74%, was "good", 25-49%, was "fair" and < 25% was "poor".

## Results

The average age of the subjects was 62.9 ± 11.2 years (57.7 ± 14.5 years for the men, 64.8 ± 9.3 for the women). The average duration of pain prior to the treatment was 68.2 ± 80.9 months (86.3 ± 133.1 months for the men, 61.7 ± 54.0 months for the women). The affected levels were L4-L5 in 31 (91.2%), L3-L4 in 19 (55.9%), L5-S1 in 15 (44.1%) and L2-L3 in 4 (11.8%) patients. Twelve (35.3%) patients had one level of stenosis, 11 (32.4%) had two levels, 9 (26.5%) had three levels and two (5.9%) had four levels (Table 1).

**Table 1:** Baseline patient characteristics

Patient	Age (yr)	Sex	Pain Duration (months)	VAS	ODI (%)	Stenosis level			
						
						L2-L3	L3-L4	L4-L5	L5-S1
1	31	M	2	8	82			○	
2	35	F	4	9	66			○	
3	36	M	140	8	42			○	
4	54	F	72	10	18			○	○
5	55	F	36	7	44			○	
6	56	F	36	10	38		○	○	
7	57	M	420	5	42			○	
8	58	F	120	6	32		○	○	
9	59	M	24	5	22		○	○	
10	59	F	8	5	32			○	
11	60	F	108	6	8	○	○		○
12	60	F	2	9	40		○	○	○
13	62	F	36	5	22			○	
14	63	F	48	6	20		○	○	
15	64	M	18	5	52			○	○
16	64	M	36	6	36		○	○	
17	65	F	18	10	50			○	
18	65	F	240	6	28		○	○	○
19	65	F	36	5	27		○	○	
20	66	F	4	5	36		○		○
21	66	F	6	8	62		○		○
22	68	M	96	5	44	○	○	○	○
23	68	M	5	10	66		○	○	○
24	69	F	60	8	30		○	○	○
25	70	F	120	9	48		○	○	○
26	70	F	92	10	54			○	○
27	71	F	80	10	40		○	○	○
28	72	M	36	5	36	○	○	○	
29	72	F	96	6	22			○	
30	73	F	36	8	30	○	○	○	○
31	76	F	120	10	60			○	
32	76	F	80	8	68			○	
33	77	F	60	6	40			○	
34	77	F	24	10	70		○	○	○

VAS, visual analogue scale pain score; ODI, the Oswestry Disability Index score.

Four (11.8%) patients received the treatment one time, 20 (58.8%) underwent two interventions, 7 (20.6%) had three interventions, two (5.9%) received four interventions and one (2.9%) patient underwent five interventions. No significant or fatal adverse effects were reported following the treatment. The average follow-up period was 12.9 ± 1.1 months.

After the patients underwent fluoroscopically guided transforaminal epidural dry needling, the mean VAS pain score decreased by 2.7 ± 3.0 points (from 7.3 ± 2.0 at baseline to 4.6 ± 2.5 at follow-up) (*p *< 0.01), the per cent disability score on the ODI decreased by 15.9 ± 19.5% (from 41.4 ± 17.2% at baseline to 25.5 ± 12.6% at follow-up) (*p *< 0.01), and the average of the self-rated improvement following the intervention was 52.6 ± 33.1% (*p *< 0.01). The VAS scores indicated that 14 (41.2%) patients reported a "good" to "excellent" treatment response, 11 (32.4%) patients had a "good" to "excellent" treatment response on the ODI and 22 (64.7%) patients had a "good" to "excellent" treatment response on the self-rated improvement scale (Table 2).

**Table 2:** Treatment responses to fluoroscopically guided transforaminal epidural dry needling

Outcome measure	Average change			Percent change			
	
	Baseline	Follow-up	*P*	Excellent (≥ 75%)	Good (50-74%)	Fair (25-49%)	Poor (< 25%)
VAS pain score (points)	7.3 ± 2.0	4.6 ± 2.5	< 0.0001	7 (20.6)	7 (20.6)	4 (11.8)	16 (47.1)
Percent disability of ODI score (%)	41.4 ± 17.2	25.5 ± 12.6	< 0.0001	7 (20.6)	4 (11.8)	6 (17.6)	17 (50.0)
Self-rated improvement following treatment (%)	-	52.6 ± 33.1	< 0.0001	10 (29.4)	12 (35.3)	4 (11.8)	8 (23.5)

VAS, visual analogue scale pain score; ODI, Oswestry Disability Index score.

## Discussion

The VAS and ODI scores improved significantly following fluoroscopically guided transforaminal epidural dry needling, and nearly 65% of the patients rated their response as "good" to "excellent" on the self-rated improvement assessment. These results suggest that fluoroscopically guided transforaminal epidural dry needling is an effective intervention for managing LSS.

The technique was developed from our clinical experience in dry needling of neural tissues. We have found that the technique has a beneficial effect on various chronic pain conditions including spinal pain, without the addition of medication [19,20]. For the treatment mechanisms of our intervention, we hypothesise that dry needling improves nerve mobility, reduces neuronal hypersensitivity and promotes the natural healing process, resulting in improvement of painful conditions.

Although we could not demonstrate the presence of adhesion between the nerve root and surrounding tissue, surgeons report that adhesion is a common pathological condition in LSS and studies demonstrate that it plays an important role in the pathogenesis of lumbar spinal disorders, including LSS [21]. We believe that dry needling into the intervertebral foramen using a 1.2-mm-diameter needle is sufficient to release the adhesion between the nerve root and surrounding tissue and thereby decompress the lumbar spinal nerve, thus improving nerve mobility and alleviating pain.

Most of the patients who were not sedated and locally anaesthetised with lidocaine reported that they experienced a sensation like an electric current running through their back or lower extremity when the needle was advanced into their epidural space. We could observe twitch responses of the muscles innervated by the affected spinal nerves in the subjects during the procedure. Based on the patients' experiences and our observation, we hypothesised that needling induces neuronal reflex, thereby reducing hypersensitivity of the neural tissue.

Lumbar spinal stenosis causes prolonged blocking of the cauda equina and nerve root(s). Studies have demonstrated that hindering neuronal impulses for a period of time causes hypersensitivity of the denervated organs [22,23]. Dry needling produces minute wounds. The wounds generate a current of injury continuously for several days or weeks, which promotes the natural healing process and a decline in denervation hypersensitivity [24,25]. Furthermore, blood flow increases around the injured area, which may relieve localised ischaemia or vasoconstriction.

**Figure 4:**
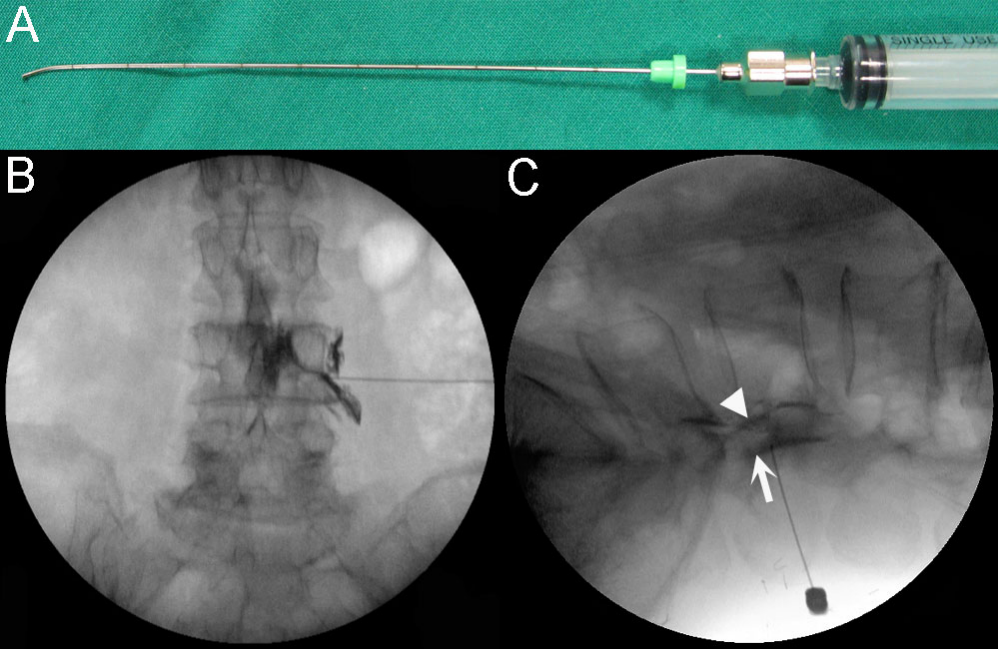
**Transforaminal epidural contrast injection test to demonstrate that the specially designed needle effectively reached the target structures**. The curved spinal injection needle used in the test (A). The anteroposterior fluoroscopy image shows a needle that has been advanced transforaminally at the L4-L5 level in the same manner as that used in the treatment and contrast agent flowing through the epidural space (B). The lateral fluoroscopy image. The triangle (▼) indicates contrast agent in the anterior epidural space when the needle was used in its original straight form. The arrow (↑) indicates contrast agent in the posterior epidural space when the curved needle was used. The curved needle contacted the canal side of the inferior articular process between the facet joint and pedicle (C).

A transforaminal epidural contrast injection test using a spinal needle was conducted to examine whether the flexed Round Needle effectively reached the posterior epidural space, the primary target of our intervention. When the spinal needle was advanced transforaminally in its original straight form, we found it difficult to contact the canal side of the inferior articular process between the facet joint and pedicle and advance further into the anterior epidural space. However, when the tip was curved (Figure 4A), one could easily contact the canal side of the inferior articular process between the facet joint and pedicle and reach the posterior epidural space (Figures 4B and 4C), indicating that the bent needle allowed us to reach the primary target of our intervention.

**Figure 5:**
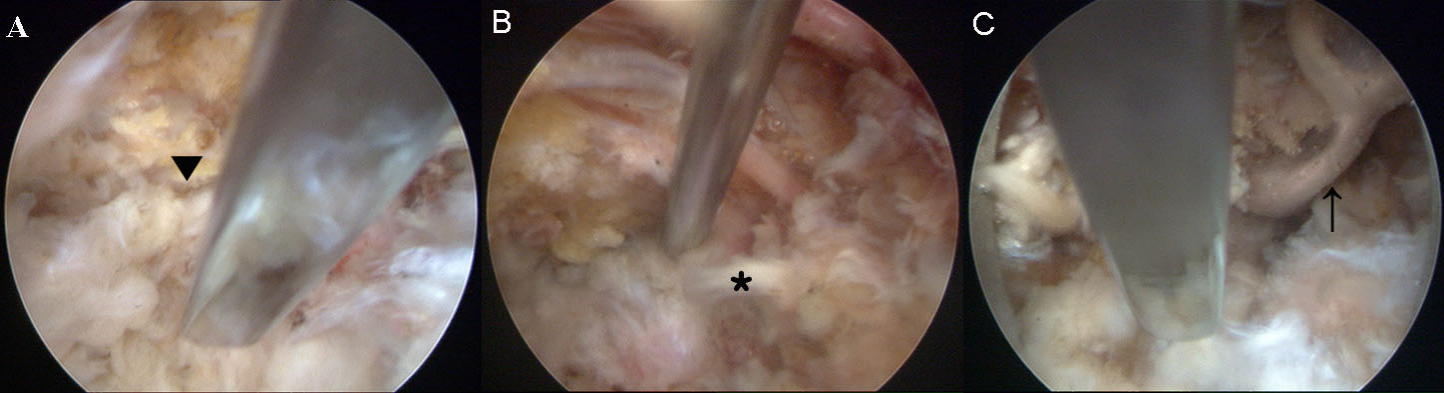
**A cadaveric examination used to evaluate the safety of fluoroscopically guided transforaminal epidural dry needling**. The flexed Round Needle contacted and passed by the facet joint (▼) is advanced into the intervertebral foramen (A). The needle passes over the posterior longitudinal ligament (*) and reaches the anterior epidural space in the same manner as with the anterior epidural approach (B). After removing the posterior longitudinal ligament and dura mater by cauterisation, the tip of the needle is found to be located under the thecal sac (↑) without contacting it (C).

While performing interventions, we were careful to avoid complications such as haematoma, dural puncture and anterior spinal artery syndrome, and no evidence of complications was detected in, or reported by, the patients. We applied the intervention clinically after confirming its safety in cadaveric examinations. Figure 5 shows a cadaveric study performed to evaluate the safety of fluoroscopically guided transforaminal epidural dry needling. The flexed Round Needle was inserted transforaminally into the right L3-L4 intervertebral foramen of a cadaver under C-arm fluoroscopic guidance. An endoscope was introduced following the same route and traced the needle (Figures 5A, B and 5C). The study demonstrated that our intervention does not injure critical spinal structures, including the spinal nerve, dura mater and thecal sac. In clinical practice, the needle primarily approaches the posterior epidural space. However, demonstrating the approach to the posterior epidural space in this cadaveric study was impossible because the epidural space was congested with the needle and endoscope, and limited space was available for J-turning the endoscope to visualise the posterior epidural space.

We believe that the streamlined shape, solid but flexible body and round blunt tip of the specially designed needle minimise tissue damage. In most cases, the needle is advanced to the outer-third of the pedicle and moved along the canal side of the inferior articular process between the facet joint and pedicle. The needle is introduced into the "safe triangle", which was suggested by Bogduk *et al*. [26] as the roof made up by the pedicle, a tangential base that corresponds to the exiting nerve root, and the lateral border of the vertebral body.

Our technique shares some aspects of conventional spinal interventions for LSS, such as transforaminal epidural steroid injection and epiduroscopic adhesiolysis. However, it has several unique features and advantages. While the conventional transforaminal epidural steroid injection approach is similar to ours, we do not use corticosteroid, which can cause collagen degeneration and disturb the healing process, although it may also reduce inflammation. The spread of corticosteroid into critical structures beyond the treatment target is rare, but it can cause serious complications [27]. Our technique uses a needle with a blunt, round tip, so the treatment effect is limited to the needling points. An epiduroscope is introduced into the sacral hiatus and advanced into the interlaminar posterior epidural space. When an adhesion or thick connective tissue is detected in the epidural space, it is broken down using saline injections and steroid or local anaesthetic [28]. The lumbar epidural space in adults is segmented and discontinuous and may impede the passage of the epiduroscope and cause its misplacement [29]. Using a needle in the transforaminal route may break down adhesion between the nerve root and surrounding tissues. It also allows access to the upper lumbar levels.

The design of this study, a case series, poses a limitation, and further studies or randomised clinical trials are needed to evaluate the efficacy of our technique compared with other treatment methods for LSS. We have suggested possible treatment mechanisms, but they are not fully supported by other studies. Further research is needed to evaluate our hypothesis, including animal studies. Although this study suggests that our technique is effective for managing LSS, fewer than half of the subjects reported a "good" to "excellent" treatment response as evaluated by the VAS and ODI scores. Further work is necessary to increase the favourable treatment response.

## Conclusions

We developed a minimally invasive interventional technique using a specially designed needle to treat LSS. Thirty-four patients with LSS underwent fluoroscopically guided transforaminal epidural dry needling. At the level of the stenosis, a specially designed flexed Round Needle was inserted 8-12 cm lateral to the midline and advanced to a position between the anterior side of the facet joint and pedicle. It was advanced medially and backed laterally along the canal side of the inferior articular process between the facet joint and pedicle. The procedure was completed when the resistance at the tip of the needle was markedly reduced. The treatment outcome was assessed using a VAS pain score, the ODI and self-rated improvement. A significant improvement was found in each measure after the procedure. These results suggest that fluoroscopically guided transforaminal epidural dry needling is an effective intervention for managing LSS.

## Competing interests

KA developed the 'Round Needle', the specially designed needle used in this study. He possesses Korean patent 10-2004-41689 on the needle. However, he has no stocks or ownership in the company manufacturing the needle and has not been compensated for possessing the patent by any individual or organisation. None of the authors receives reimbursements, fees, funding or salary from any individual or organisation related to the content of the manuscript.

## Authors' contributions

KA developed the fluoroscopically guided transforaminal epidural dry needling technique and the specially designed needle used in this study. HJJ analysed the data and wrote the manuscript. TKL and YSL collected the data. All authors read and approved the final manuscript.

## Pre-publication history

The pre-publication history for this paper can be accessed here:

http://www.biomedcentral.com/1471-2474/11/180/prepub

## Supplementary Material

Additional file 1 **Windows Media Video**. Fluoroscopically guided transforaminal epidural dry needling for lumbar spinal stenosis using a specially designed needle. A movie demonstrating the technique Click here for file
